# Developing a Research Agenda and a Comprehensive National Prevention and Response Plan for Rift Valley Fever in the United States

**DOI:** 10.3201/eid1308.070551

**Published:** 2007-08

**Authors:** Seth C. Britch, Kenneth J. Linthicum, the Rift Valley Fever Working Group

**Affiliations:** * US Department of Agriculture–Agricultural Research Service, Gainesville, Florida, USA

**Keywords:** Rift Valley fever, research needs, surveillance, control, prevention, conference summary

The 1999 introduction and rapid spread of West Nile virus (WNV) across the United States demonstrated our vulnerability to emerging mosquito-borne viruses, but also showed the abilities of the US public health and animal health systems to identify weaknesses and develop strategies to reduce them. For example, although many counties already had vector and disease surveillance programs, the arrival of WNV compelled more communities to act. Unfortunately, the protective infrastructure that grew during our reaction to WNV is dissolving as funding is repositioned to new threats. With each new threat, public health and animal health agencies are charged with developing response plans and disbursing funds to a tangled web of bench scientists and “boots on the ground.” Two key shortfalls in our approach to emerging disease threats have come out of this: 1) US public health and animal health agencies have become reactive rather than proactive—new committees and infrastructure are formed to deal with new threats and wheels are reinvented; and 2) because agencies tend to work independently, wheels are reinvented in parallel.

Given these shortfalls, on December 5, 2006, the Animal and Plant Health Inspection Service (APHIS) Centers for Epidemiology and Animal Health in Fort Collins, Colorado, hosted a multi-agency and university working group to confront the issue of Rift Valley fever (RVF), a mosquito-borne zoonotic hemorrhagic viral disease confined mainly to sub-Saharan Africa. In these countries, human and livestock populations bear prominent health and economic effects during an RVF outbreak (*1*). Sheep, goats, and cattle are particularly susceptible to the disease. RVF virus (RVFV) is classified as an overlap select agent, i.e., affecting both humans and non-human animals, by both the Centers for Disease Control and Prevention (CDC) and APHIS (*2**,**3*). If introduced into the United States RVFV could be spread by mosquitoes like WNV is, but could also be spread by contact with infected vertebrate tissues or aerosols (*4*). No approved human or animal vaccines exist for use in the United States.

The Rift Valley Fever Working Group comprises >30 participants from more than a dozen US government agencies and universities. It was launched by scientists from the US Department of Agriculture-Agricultural Research Service (USDA-ARS) and the University of Wyoming during a smaller spring meeting in 2006. At that meeting, participants discussed RVF research, ranging from vaccine and diagnostics development to spatially explicit geographic information system (GIS)–based modeling of potential US vector mosquitoes, as well as new collaborative initiatives focused on the risk for mosquito transmission of RVFV after natural or intentional introduction. The capstone of the spring meeting was the development of an outline for an interagency RVF research and response group. This led to the winter meeting with over 20 presentations and facilitated discussions covering a broad scope of RVF issues. Several presentations highlighted areas of research that would improve our response to RVF, for example, ecologic modeling of vectors; communications and reporting models; surveillance, response, and control models; and vaccine and diagnostics development. Many presentations showed how individual agencies have responded to RVF in the past, and recommendations for future collaborations were discussed. The group also detailed how other emerging infectious disease (EID) response plans or unique agency services could be applied to RVF outbreaks.

Three recommendations surfaced from the winter meeting: 1) mine and synthesize existing surveillance and control plans developed for other mosquito-borne pathogens; 2) identify research and infrastructure needs unique to RVF, especially methods of RVFV transmission in the United States among mosquitoes, wildlife, and livestock; and 3) work as a multiple agency and university coordinating and coordinated group.

The first presentations and discussions detailed existing animal health surveillance systems, including probability-based surveillance, applications of GIS, spatial analysis, and remote sensing, and an existing analysis of pathways and vulnerabilities where RVFV could enter the United States. One of the first observations was that despite many EID surveillance and control plans throughout government agencies, no effective action plans are in place. Most EID response plans did not clearly identify the action agencies responsible for synthesizing information, declaring an emergency, and implementing the plan. Plans were structured to depend upon coordinated action from an array of agencies that were not necessarily aware of being part of a response plan. In addition, a substantial latent overlap among plans at multiple levels would likely lead to confusion and delay if simultaneously implemented. Given the potential conflict of procedures between a federal agency directing an emergency response and state and local agencies expected to implement, manage, and equip response actions, the group recommended that service providers (e.g., manufacturers of personal protective equipment, veterinarians, public information officers, or aerial insecticide contractors) and stakeholders (e.g., livestock producers) be involved during plan development. For instance, the United States has no capability for a national response to a vector-borne disease. The United States has national and state assets for vector surveillance, but none for vector control. In the event of an RVF outbreak, we would have to rely on cooperation from local mosquito abatement agencies that may or may not be distributed where they are most needed. The US military has the logistic capability to perform vector control anywhere in the country and has done so on a case-by-case basis, but no agreements or even discussions have taken place to make the military part of a vector-borne disease response plan.

The second round of discussions and presentations exposed critical gaps in our knowledge of RVFV transmission—from field experiences with RVF and its vectors to information about affected humans and animals in RVF-endemic regions of Africa and the Arabian Peninsula. A huge gap was the near absence of veterinary surveillance and the tools to perform it safely. Early discussions showed that more epidemiologists and entomologists should be in the field to better characterize endemic disease patterns and analyze outbreaks in real time, eventually reducing their impact. A major obstacle in studying RVF transmission is the lack of early detection—the lag time between the outbreak and the limited time in which infected vectors and vertebrates can be collected and analyzed. The Department of Defense–Global Emerging Infections System/National Aeronautics and Space Administration (DoD-GEIS/NASA) remote-sensing model that forecasts outbreaks of RVF in Africa (*5**,**6*) was discussed in detail, since at the time of the meeting the model indicated that conditions in Africa were favorable for an outbreak within 1–2 months (*7*). The value and accuracy of this model were proven shortly thereafter. The RVF disease outbreak reported December 20, 2006, in Garissa, northeastern Kenya (*8*), occurred in an east African zone flagged by the model in November 2006 as a region at high risk for RVFV transmission (*7*). Due in part to this early warning, several meeting attendees were able to respond swiftly to the winter 2006/2007 RVF outbreak by traveling to affected areas and carrying out research early in 2007. This timely response will hopefully strengthen the justification for future research funding into all aspects of the epidemiology of RVFV and its potential threat to the United States. In this session, all participants agreed that more data are needed on differences among mosquito species and mosquito populations in Africa, particularly with respect to vector competence, host preference, and transovarial transmission of RVFV. Should RVF virus be introduced to the United States, we would need similar information on US mosquitoes and other potential vectors. Detailed information on vectorial capacity, adjusted according to location and season, would be a valuable tool for prioritizing veterinary and entomologic interventions. Information is limited on the possible role of wildlife in the maintenance and amplification of RVFV in Africa, and no information is available on the role of wildlife in the United States. Of particular note, attendees discussed the absence of a US licensed veterinary vaccine for RVFV and the limited availability of reagents and vaccinated staff to perform RVF diagnosis. Efforts are now under way across several agencies to accelerate vaccine and diagnostics discovery.

Many attendees expressed concern about the potential dynamics of RVF among vectors, wildlife, livestock, and humans should the virus arrive in the United States. Of particular concern was the familiar biogeographic overlap of the habitat of potentially susceptible vertebrate hosts seen throughout the United States such as feedlots, housing tracts, and woodlands. One scenario put forth was that RVFV could arrive in the United States in imported, infected mosquitoes on ships or airplanes. Another possibility would be importation by air of a seemingly healthy vertebrate animal (or human) that subsequently develops a viremia sufficient to infect US vector mosquitoes. The infected mosquitoes could quickly spread RVFV to nearby livestock or wildlife. RVF incubation and viremia are short, and once in a natural environment. RVF could escape detection indefinitely unless observers serendipitously encountered it or were dispatched specifically to search for indicators such as unusually high levels of abortions among deer, elk, or wild sheep. Once RVFV enters populations of US vector mosquitoes, the virus could become endemic unless the introduction is detected early and action agencies are able to respond quickly and appropriately. Conference participants also stressed the importance of training producers, wildlife and livestock health professionals, wildlife biologists, and public health professionals to recognize the early signs of an RVF epizootic or epidemic. Attendees noted a clear deficit in the availability of Biosafety Level (BSL)–3 laboratories, which require RVF virus vaccinated staff, and BSL-4 laboratories, which require large animal isolation facilities to safely conduct research and develop an effective RVF response plan. Research needs include determining the susceptibility of US mosquito species, domestic livestock, and wildlife to RVF. Compounding the risk assessment for vulnerable wildlife and livestock are what attendees described as vast exotic ruminant wildlife operations in Texas that are largely unregulated. Add to this the surprising unavailability of maps of all livestock operations across the United States, due in large part to reluctance of producers to provide information on livestock populations at the necessary spatial scale and detail. Attendees from one agency described a GIS model they developed to estimate locations of livestock operations and how it could be integrated into quarantine and incident management activities.

In summary, the working group concluded that reactive and fragmentary implementation will compromise even the most sophisticated RVF prevention and response plans. Attendees were determined to synthesize expertise, coordinate research to minimize overlap, align objectives, and keep interagency dialogue on RVF open and dynamic. By meshing abilities and expectations across agencies at the planning phase, attendees believed implementation of an RVF comprehensive prevention and response plan would be realistic. One of the goals was that aspects of the RVF prevention and response plan become universal for mosquito-borne viruses and be applicable to groups concerned with other EIDs. In keeping with structuring a national comprehensive and all-agency RVF response plan, the winter meeting ended with intensive discussions on enlisting additional collaborators, partners, and speakers for future meetings. By mid-2007, the RVF Working Group will produce a draft white paper detailing RVF background, research needs, action contributions from member agencies and universities, surveillance and control needs, and RVF prevention and preparation outlines for the United States.

## References

[1] Megan JM, Bailey CL. Rift Valley fever. In*:* The arboviruses: epidemiology and ecology, vol IV. Boca Raton (FL): CRC Press; 1989. p. 51–76.

[2] Department of Agriculture, Animal and Plant Health Inspection Service: Agricultural Bioterrorism Protection Act of 2002; Possession, Use, and Transfer of Biological Agents and Toxins. 7 CFR part 331 and 9 CFR part 121; 2005.

[3] Department of Health and Human Services. Possession, Use, and Transfer of Biological Agents and Toxins, 42 CFR parts 72 and 73; 2005.

[4] House JA, Turell MJ, Mebus CA. Rift Valley fever—present status and risk to the Western Hemisphere. Ann N Y Acad Sci. 1992;653:233–42. 10.1111/j.1749-6632.1992.tb19652.x 1626877

[5] Linthicum KJ, Anyamba A, Tucker CJ, Kelley PW, Myers MF, Peters CJ. Climate and satellite indicators to forecast Rift Valley fever epidemics in Kenya. Science. 1999;285:397–400. 10.1126/science.285.5426.397 10411500

[6] Department of Defense–Global Emerging Infections System. [cited 2007 Jun 27]. Available from: http://www.geis.fhp.osd.mil/GEIS/SurveillanceActivities/RVFWeb/indexRVF.asp

[7] Anyamba A, Chretien JP, Small J, Tucker CJ, Linthicum KJ. Developing global climate anomalies suggest potential disease risks for 2006–2007. Int J Health Geogr. 2006;5:60 . 10.1186/1476-072X-5-60 17194307 PMC1779293

[8] Kenya: five more die from unidentified disease., East African Standard (Nairobi) 2006 Dec 20. [cited 2007 Jun 27]. Available from http://allafrica.com/publishers.html?passed_name=The%20East%20African%20Standard&amp;passed_location=Nairobi

